# MOVING BEYOND INDIVIDUAL COPING TO DEVELOPING SYSTEMS-LEVEL INTERVENTIONS FOR COMMUNITY-DWELLING ADULTS

**DOI:** 10.1093/geroni/igz038.2960

**Published:** 2019-11-08

**Authors:** Terri Lewinson, Sharon Bowland

**Affiliations:** 1 Georgia State University, School of Social Work, Atlanta, Georgia, United States; 2 University of North Texas, Denton, Texas, United States

## Abstract

According to AARP, the number of Americans aged 65 and older will grow to 90.5 million, which is double the count in 2010. Along with the growth of the aging population, we can also anticipate more diverse older adult communities due to increased immigration and evolving, complex family constellations. Two major concerns for aging adults is managing increasing geriatric conditions that affect mobility/function and chronic morbidities, while also trying to afford quality housing, the environmental context within which older adults spend most of their time. Urban planners and human service providers must remain abreast of the housing-health needs of this aging population and prepare aging-friendly communities that foster resident resilience and address “environmental, economic, and social factors that influence the health and wellbeing of older people” (AARP, 2017). In this symposium, panelists one present findings from a qualitative study to explore collective trauma and resilience narratives among residents in Flint, Michigan. Panelists two share findings from a community-based participatory study that explores the intricate network of relationships among residents as a core indicator of livability in senior housing. Panelists three share strategies for strengthening networks to reducing resident isolation and loneliness. Lastly, the fourth set of panelists discuss policy implications and trauma-informed approaches needed for residents with a history of interpersonal trauma.

